# Inhibition of JNK-mediated autophagy enhances NSCLC cell sensitivity to mTORC1/2 inhibitors

**DOI:** 10.1038/srep28945

**Published:** 2016-06-30

**Authors:** Hyeon-Ok Jin, Sung-Eun Hong, Jin-Ah Park, Yoon Hwan Chang, Young Jun Hong, In-Chul Park, Jin Kyung Lee

**Affiliations:** 1 KIRAMS Radiation Biobank, Korea Institute of Radiological and Medical Sciences, 75 Nowon-ro, Nowon-gu, Seoul, 01812, Republic of Korea; 2 Division of Radiation Cancer Research, Korea Institute of Radiological and Medical Sciences, 75 Nowon-ro, Nowon-gu, Seoul, 01812, Republic of Korea; 3 Department of Laboratory Medicine, Korea Cancer Center Hospital, Korea Institute of Radiological and Medical Sciences, 75 Nowon-ro, Nowon-gu, Seoul, 01812, Republic of Korea

## Abstract

As the activation of autophagy contributes to the efficacy of many anticancer therapies, deciphering the precise role of autophagy in cancer therapy is critical. Here, we report that the dual mTORC1/2 inhibitors PP242 and OSI-027 decreased cell viability but did not induce apoptosis in the non-small cell lung cancer (NSCLC) cell lines H460 and A549. PP242 induced autophagy in NSCLC cells as demonstrated by the formation of massive vacuoles and acidic vesicular organelles and the accumulation of LC3-II. JNK was activated by PP242, and PP242-induced autophagy was blocked by inhibiting JNK pathway with SP600125 or JNK siRNA, suggesting that JNK activation is required for the mTORC1/2 inhibitor-mediated induction of autophagy in NSCLC cells. Inhibiting JNK or autophagy increased the sensitivity of H460 cells to mTORC1/2 inhibitors, indicating that JNK or autophagy promoted survival in NSCLC cells treated with mTORC1/2 inhibitors. Together, these data suggest that combining mTORC1/2 inhibitors with inhibitors of JNK or autophagy might be an effective approach for improving therapeutic outcomes in NSCLC.

Autophagy is an evolutionarily conserved catabolic degradation process by which cellular proteins and organelles are engulfed by autophagosomes, digested in lysosomes, and recycled in order to sustain cellular homeostasis^1^. Autophagy can function as a cellular housekeeper by removing damaged organelles and recycling macromolecules; therefore, autophagy can protect cancer cells, particularly during malignant transformation and carcinogenesis. A number of studies have indicated that autophagy is stimulated under conditions of starvation and hypoxia by various tumor cell survival mechanisms and that inhibiting autophagy decreases tumor growth^2^,^3^. In addition, autophagy is upregulated in tumor cells treated with chemotherapeutic agents, thereby enhancing drug resistance and decreasing the anti-cancer effects of chemotherapy^4^,^5^. In clinical models, inhibiting pro-survival autophagy using genetic or pharmacological means has been shown to kill tumor cells and trigger apoptotic cell death^6^,^7^,^8^,^9^,^10^,^11^. Therefore, targeting autophagy is considered a promising therapeutic strategy for treating cancer.

The mammalian target of rapamycin (mTOR) is a master regulator that integrates cues from external and internal signals, such as growth factors, amino acids, glucose and energy status to control growth and metabolism^12^,^13^. mTOR consists of 2 distinct complexes, referred to as mTORC1 (composed of mTOR, GβL and raptor) and mTORC2 (composed of mTOR, GβL, rictor and SIN1). mTORC1 is primarily involved in protein synthesis and cellular metabolism, and mTORC2 plays an important role in the regulation of the cytoskeleton. mTORC1 suppresses autophagy by phosphorylating the autophagy-associated kinases ULK1 and ULK2^1^,^14^,^15^. mTORC2 inhibits autophagy by phosphorylating AKT^16^. Increased autophagic activity is frequently observed in malignant cells in response to treatment with mTOR inhibitors^17^, and this effect has been hypothesized to significantly reduce treatment efficacy.

In this study, we observed that mTORC1/2 inhibitors induce cytoprotective autophagy by activating JNK in NSCLC cells and that inhibiting autophagy or JNK activation increases NSCLC cell sensitivity to mTORC1/2 inhibitors. Therefore, combination treatment with mTORC1/2 inhibitors and inhibitors of JNK or autophagy might be a promising approach to improving therapeutic outcomes in NSCLC.

## Results

### PP242 and OSI-027 inhibit mTORC1 and mTORC2 signaling and reduce cell viability

**Figure 1:**
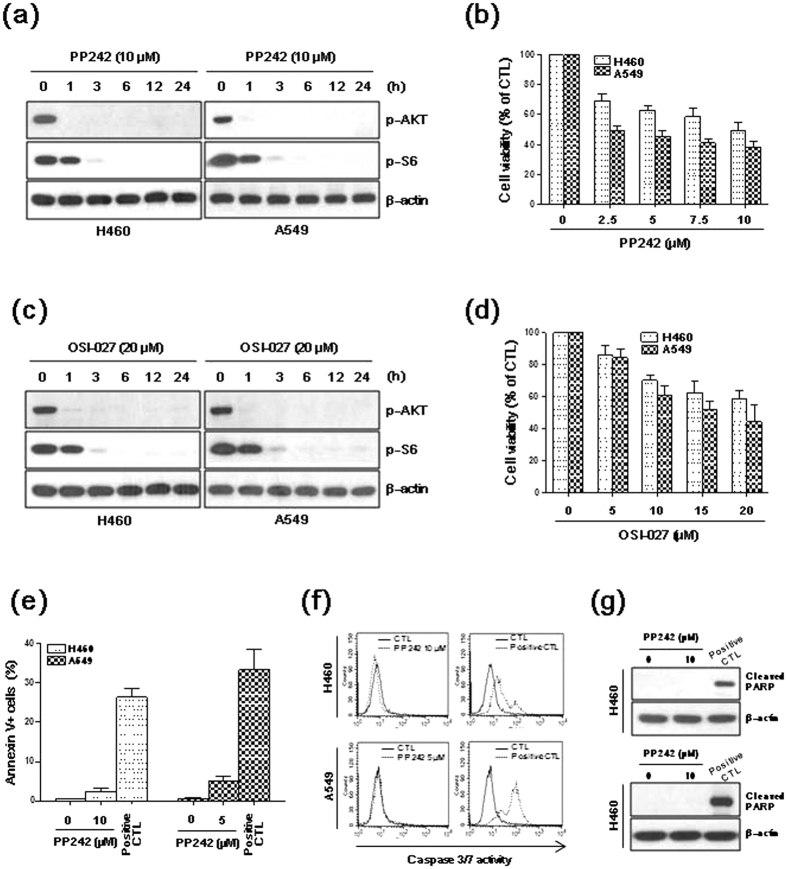
Effects of mTORC1/2 inhibitors on NSCLC cells. (**a–d**) H460 and A549 cells were treated with 10 μM PP242 or 20 μM OSI-027 for the indicated amount of time. (**e–g**) H460 or A549 cells were treated with 10 μM or 5 μM PP242, respectively, for 24 h. (**a**,**c**,**g**) Protein levels were estimated using western blot analysis. The blot shown is representative of 3 independent experiments. (**b,d**) Cell viability was measured using the MTT assay. The data are presented as the mean percentage of control ± SD (n = 3). (**e**) Apoptosis was measured as the percentage of annexin V-positive cells. (**f**) Caspase-3/7 activity was evaluated using a CaspaTag *in situ* Assay Kit. Data representative of 2 independent experiments are shown. (**e,f**) H460 and A549 cells treated with 10 μM lapatinib/100 nM bafilomycin A1 and 10 μM lapatinib/5 nM bafilomycin A1, respectively, for 24 h were used as positive controls. CTL, control.

We first investigated mTORC1/2 activity in lung cancer cells treated with the mTORC1/2 inhibitors PP242 or OSI-027. H460 and A549 cells were treated with PP242 (10 μM) or OSI-027 (20 μM) for various periods of time, and the phosphorylation of S6 (a downstream effector of mTORC1) and AKT (a downstream effector of mTORC2) were analyzed using western blot. PP242 and OSI-027 inhibited the phosphorylation of S6 and AKT in a time-dependent manner (Fig. 1a,c). Next, we investigated the effects of mTORC1/2 inhibitors on cell viability. The MTT assays demonstrated that exposure to PP242 or OSI-027 for 24 h decreased the viability of H460 and A549 cells in a dose-dependent manner (Fig. 1b,d). To determine if the PP242-induced decrease in cell viability was mediated by apoptosis, H460 and A549 cells were treated with 10 μM and 5 μM of PP242, respectively, for 24, after which point, cell viability was reduced by approximately 50%. The cells were then stained with FITC-conjugated annexin V and analyzed using flow cytometry. Only 3% of H460 cells and 5% of A549 cells treated with PP242 were apoptotic (Fig. 1e), and neither caspase 3/7 activity nor PARP cleavage was observed (Fig. 1f,g). These data suggest that apoptosis is not associated with the reduction in NSCLC cell viability induced by mTORC1/2 inhibitors.

### PP242 induces autophagy in NSCLC cells

**Figure 2:**
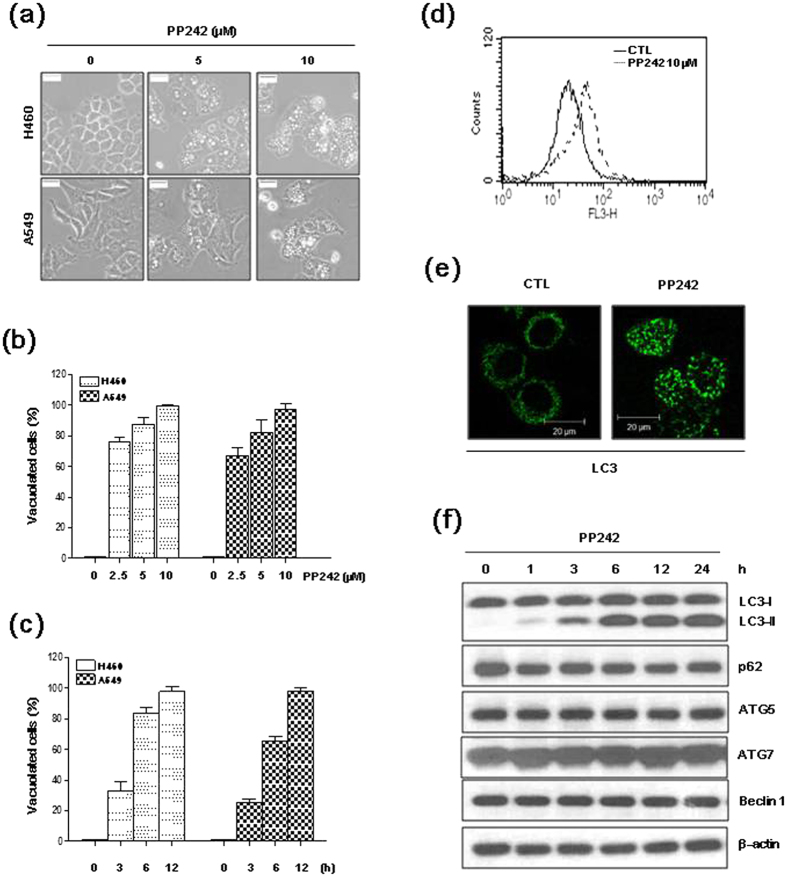
PP242 induces the formation of massive vacuoles and increases LC3-II levels in NSCLC cells. (**a,b**) H460 and A549 cells were treated with the indicated concentrations of PP242 for 24 h. (**c**) H460 and A549 cells were treated with 10 μM PP242 for the indicated amount of time. (**a**) Changes in cellular morphology were observed using an inverted microscope (magnification = 400×, scale bar = 5 mm). (**b,c**) The percentage of vacuolated cells was calculated from 3 independent images. The data are presented as the mean ± standard deviation. (**d**) H460 cells were treated with vehicle or 10 μM PP242 for 16 h, stained with acridine orange, and red fluorescence (FL3 channel) was quantified using FACS analysis. (**e**) H460 cells treated with vehicle or 10 μM PP242 for 8 h were stained for LC3 antibody after para-formaldehyde fixation. (**f**) H460 cells were treated with 10 μM PP242 for the indicated amount of time. Protein levels were estimated using western blot analysis. β-actin was used as a loading control. The blot shown is representative of 2 independent experiments.

Interestingly, extensive cellular vacuolation was observed in H460 and A549 cells treated with PP242 (Fig. 2a–c). The vacuoles became visible after approximately 3 h of PP242 treatment and became larger and denser in a time-dependent manner. As the accumulation of vacuoles is a morphological characteristics of autophagy^18^, we assessed the ability of PP242 to induce autophagy in NSCLC cells. Flow cytometry analysis demonstrated that the red fluorescence signal increased in PP242-treated H460 cells stained with acridine orange (Fig. 2d), indicating the presence of intracellular acidification, a hallmark of autophagy. On autophagic activation, LC3-I is cleaved, lipidated, and inserted as LC3-II into autophagosome membrane. In H460 cells with PP242, LC3 puncta close to the vacuoles were detected (Fig. 2e) and a marked increase in LC3-II was observed in a time-dependent manner (Fig. 2f), indicating that association of LC3 with autophagosome membranes.

Next, we measured the expression levels of other autophagy markers, including Atg5, Atg7, Beclin 1, and p62 sequestosome 1 (SQSTM1), in PP242-treated cells. The expression level of the autophagy-associated genes Atg5, Atg7, and Beclin 1 generally reflects their level of autophagic activity^19^, and the accumulation of p62 represents impaired autophagy^20^. The expression levels of Atg5, Atg7, Beclin 1, and p62 protein were not affected by PP242 (Fig. 2f).

**Figure 3:**
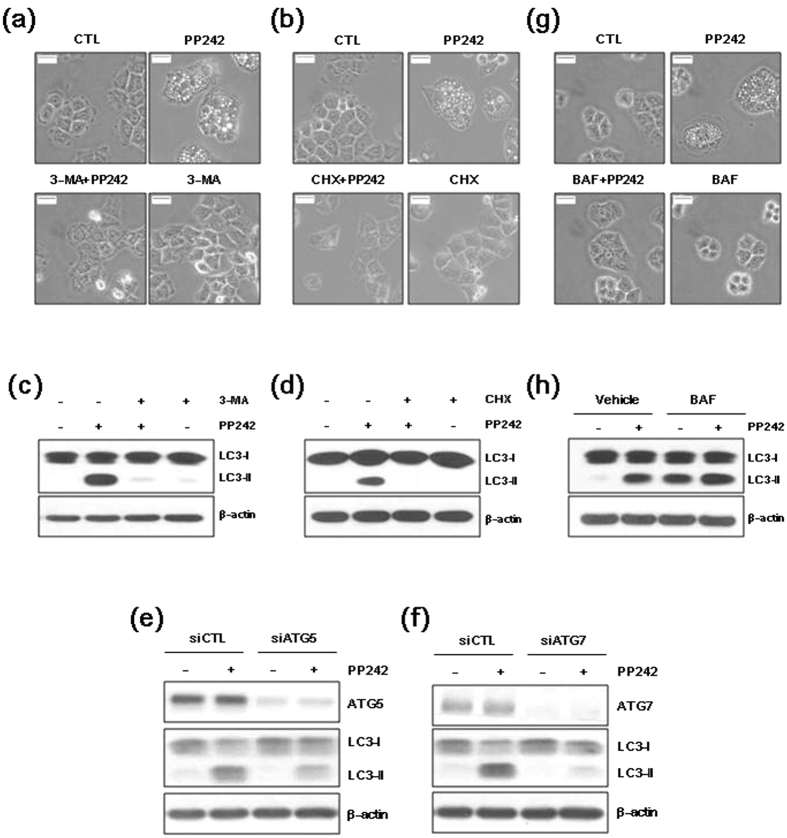
PP242 induces autophagic flux in NSCLC cells. (**a–d,g,h**) H460 cells were pretreated with 50 mM 3-MA, 20 μM CHX or 100 nM BAF for 1 h prior to treatment with 10 μM PP242 for 6 h. (**e,f**) H460 cells were transfected with control, Atg5, or Atg7 siRNAs for 16 h, and subsequently treated with 10 μM PP242 for 8 h. (**a,b,g**) Changes in cellular morphology were observed using an inverted microscope (magnification = 400×, scale bar = 5 mm). (**c–f,h**) The levels of the indicated proteins were estimated using western blot analysis. β-actin was used as a loading control. The blot shown is representative of 2 independent experiments.

We further investigated whether pharmacological inhibitors of autophagy or knockdown of Atg genes can affect autophagy induced by PP242. 3-methyladenine (3-MA; a PI3K inhibitor) and cycloheximide (CHX; an inhibitor of protein biosynthesis) are inhibitors of the early stages of autophagy^21^. Atg5 and Atg7 genes are essential for phagophore formation and ablation of either gene abrogates autophagy^22^. Pretreatment with 3-MA or CHX blocked PP242-induced vacuole formation, and significantly reduced the PP242-induced increase in LC3-II levels compared with the control cells (Fig. 3a–d). Knock-down of Atg5 or Atg7 by siRNAs blocked LC3-II expression induced by PP242 (Fig. 3e,f).

Bafilomycin A1 (BAF), a vacuolar H+-ATPase inhibitor, inhibits the later stages of autophagy by preventing lysosome function, thereby blocking the degradation of autophagosomes^21^. As shown in Fig. 3g, pretreatment with BAF blocked PP242-induced vacuole formation. LC3-II is degraded in lysosomes during autophagy; however, LC3-II can accumulate in lysosomes if protein degradation is inhibited. As shown in Fig. 3h, treatment with BAF alone increased LC3-II accumulation in vacuoles, indicating that BAF treatment blocked LC3-II degradation. The levels of LC3-II increased in cells treated with a combination of PP242 and BAF compared with cells treated with PP242 alone. These findings suggest that PP242 activates autophagic flux.

### JNK contributes to PP242-induced autophagy

**Figure 4:**
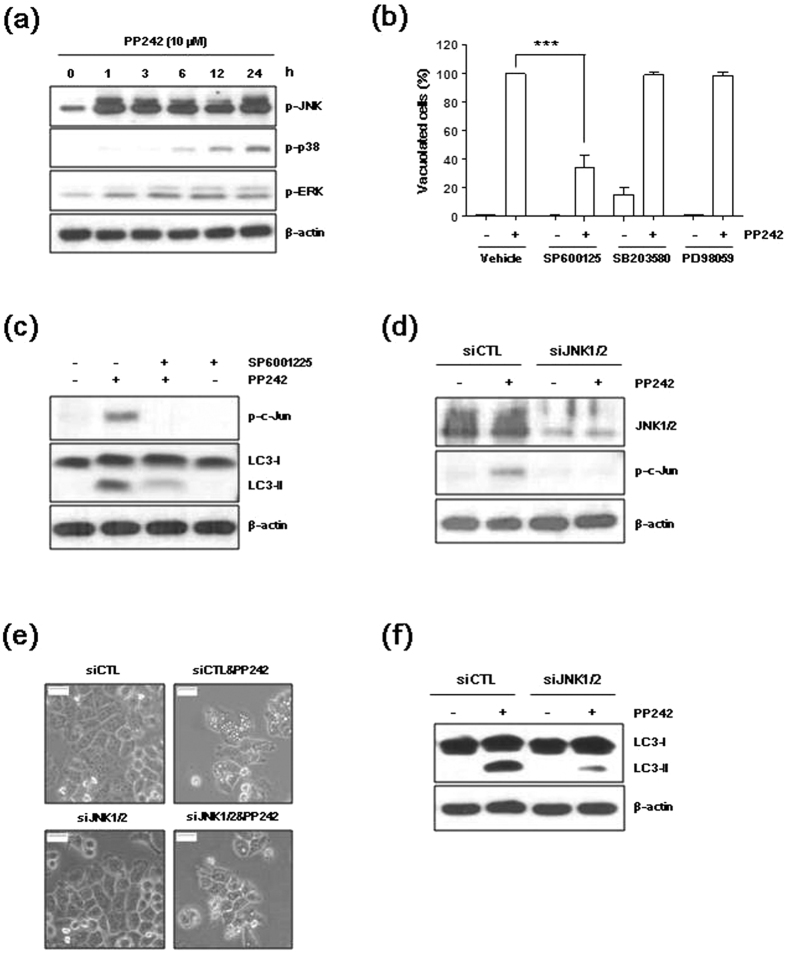
PP242 induces autophagy by activating JNK. (**a**) H460 cells were treated with 10 μM PP242 for the indicated amount of time. (**b**) H460 cells were pretreated with 20 μM SP600125 (JNK inhibitor), 20 μM SB203580 (p38 inhibitor) or 20 μM PD98059 for 1 h prior to treatment with 10 μM PP242 for 12 h. (**c**) H460 cell were pretreated with 20 μM SP600125 for 1 h and then treated with 10 μM PP242 for 24 h. (**d–f**) H460 cells were transfected with control or JNK1/2 siRNAs for 16 h, and subsequently treated with 10 μM PP242 for 24 h. (**a**,**c**,**d**,**f**). The levels of the indicated proteins were estimated using western blot analysis. β-actin was used as a loading control. The blot shown is representative of 2 independent experiments. (**b**) The percentage of vacuolated cells was calculated from 3 independent images. The data are presented as the mean ± standard deviation (n = 3, ***p < 0.001). (**e**) Changes in cellular morphology were observed using an inverted microscope (magnification = 400×, scale bar = 5 mm).

We investigated the contribution of the MAPK family protein members JNK, p38, and ERK in PP242-induced autophagy. As shown in Fig. 4a, PP242 stimulated the phosphorylation of JNK, p38, and ERK in a time-dependent manner in H460 cells. To further determine if these kinases play a role in PP242-induced autophagy, H460 cells were pretreated with SP600125 (JNK inhibitor), SB203580 (p38 inhibitor) or PD98059 (ERK inhibitor) for 1 h prior to treatment with PP242 for 12 h. As shown in Fig. 4b, PP242-induced vacuole formation was inhibited when pretreatment with SP600125. Pretreatment of SP600125 also attenuated PP242-induced c-Jun phosphorylation and LC3**-**II expression (Fig. 4c, Supplementary Fig. S3). However, no significant effect on PP242-induced vacuole formation was observed in cells pretreated with PD98059 and SB203580 (Fig. 4b). In addition, SB203580 treatment induced vacuole formation (Fig. 4b), an observation consistent with a previous report that SB203580 induced vacuole formation in HT29 human colorectal adenocarcinoma cells.

To further determine the role of JNK in PP242-induced autophagy, we transfected cells with siRNAs targeting JNK and treated them with PP242. As shown in Fig. 4d, the siRNAs targeting JNK markedly reduced the levels of JNK expression. Knockdown of JNK blocked c-Jun phosphorylation, vacuolation formation and LC3**-**II expression induced by PP242 (Fig. 4d–f). These data indicate that JNK pathway potentially plays a role in regulating autophagy in NSCLC cells treated with mTORC1/2 inhibitors.

### Reversibility of vacuolation and LC3-II accumulation

**Figure 5:**
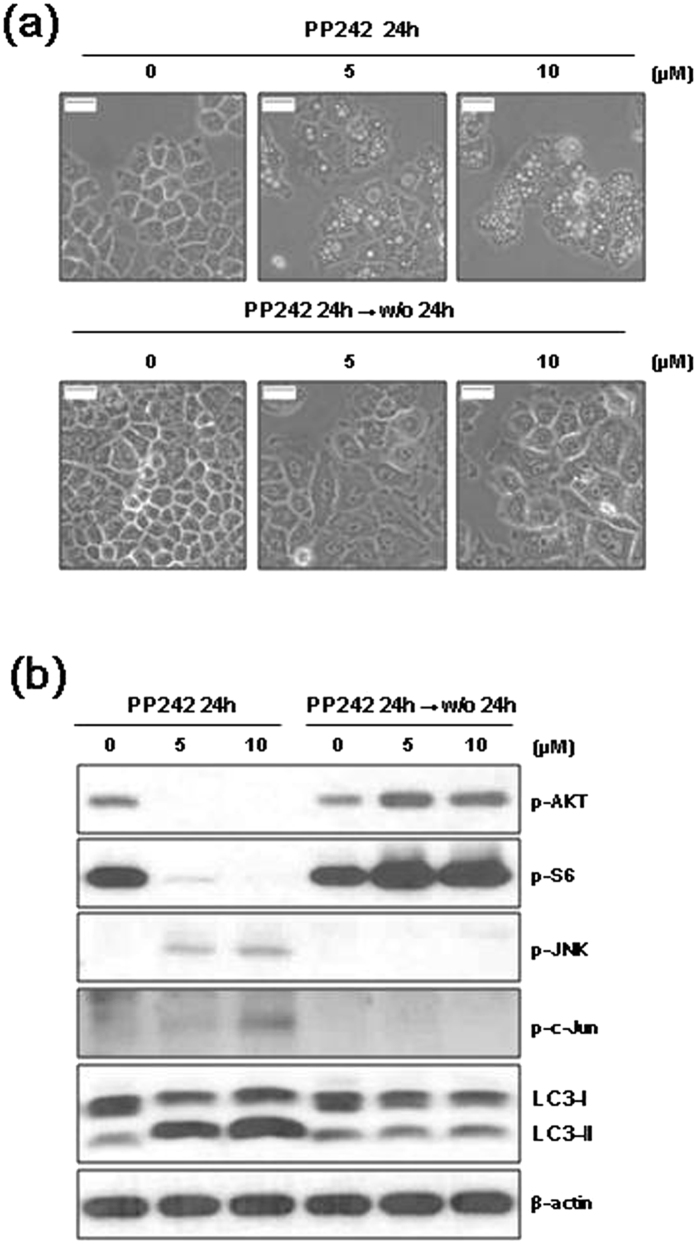
Autophagy induced by PP242 is reversed by PP242 washout. (**a**,**b**) H460 cells were treated with the indicated concentration of PP242 for 24 h before the drug was washed off and the cells were incubated for an additional 24 h. (**a**) Changes in cellular morphology were observed using an inverted microscope (magnification = 400×, scale bar = 5 mm). (**b**) The levels of the indicated proteins were estimated using western blot analysis. β-actin was used as a loading control. The blot shown is representative of 2 independent experiments.

We investigated if PP242-induced autophagy can be reversed after removing PP242 by thoroughly washing the treated cells. H460 cells were treated with 5 or 10 μM PP242 for 12 h and then incubated for 12 h in drug-free media. After PP242 was removed from the cells, the vacuoles diminished in size and disappeared within 12 h (Fig. 5a). A decrease in the levels of JNK and c-Jun phosphorylation and LC3-II expression was also observed after PP242 washout (Fig. 5b). In addition, the PP242-induced decrease in phosphorylated AKT and S6 was rescued after PP242 was removed (Fig. 5b).

### Inhibiting autophagy or JNK enhances the mTORC1/2 inhibitor-mediated reduction in NSCLC cell viability

**Figure 6:**
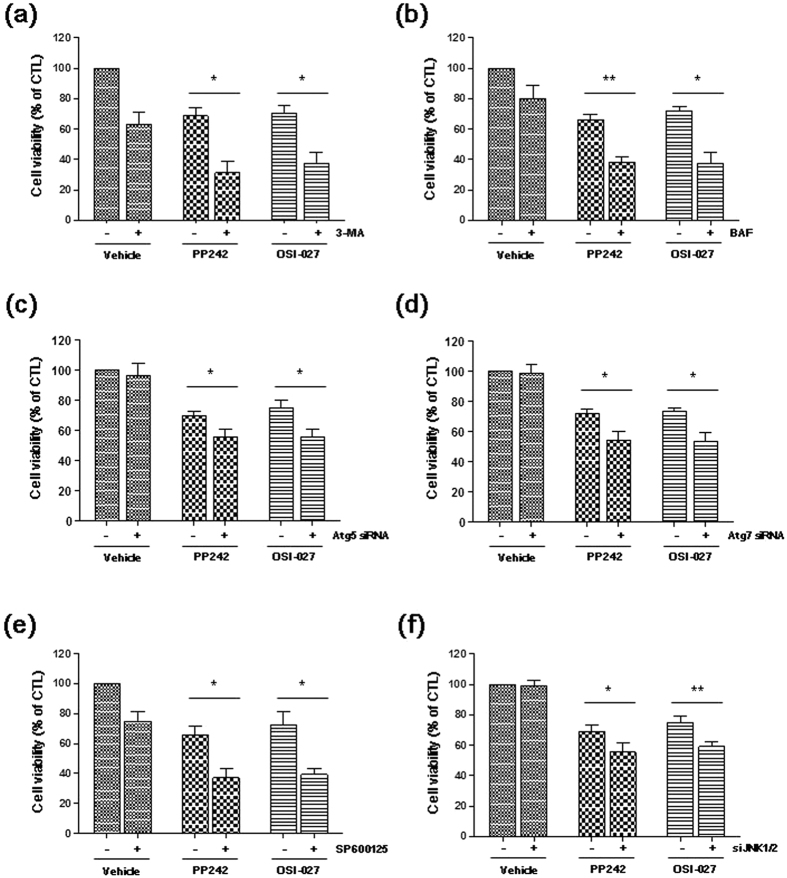
Inhibiting autophagy or JNK enhances the PP242-mediated decrease in NSCLC cell viability. (**a,b,e**) H460 cells were pretreated with 50 mM 3-MA, 100 nM BAF or 20 μM SP600125 for 1 h prior to treatment with 5 μM PP242 or 10 μM OSI-027 for 24 h. (**c,d,f**) H460 cells were transfected with control, Atg5, or Atg7, or JNK1/2 siRNAs for 16 h, and subsequently treated with 5 μM PP242 or 10 μM OSI-027 for 24 h. Cell viability was measured using the MTT assay. The data are presented as the mean percentage of control ± SD (n = 3, **p < 0.01, *p < 0.05).

We next evaluated the role of mTORC1/2 inhibitor-induced autophagy on NSCLC cells. We found that inhibition of autophagy by 3-MA or BAF enhanced the PP242 or OSI-027-mediated decrease in H460 cell viability (Fig. 6a,b). Knock-down of Atg5 or Atg7 by siRNAs also enhanced cell sensitivity to mTORC1/2 inhibitors (Fig. 6c,d). We then investigated if the autophagy promoted by JNK activation in NSCLC cells treated with mTORC1/2 inhibitors exerted a pro-survival effect. We found that inhibiting JNK by pretreating the cells with SP600125 or JNK1/2 siRNAs potentiated the cytotoxic effect of PP242 or OSI-027 in H460 cells (Fig. 6e,f). These results indicate that blocking autophagy or JNK enhances the mTORC1/2 inhibitor-induced reduction of cell viability.

## Discussion

mTOR is a downstream mediator in the PI3K/AKT signaling pathway that functions as a key regulatory protein in cell growth and proliferation^23^,^24^. Dysregulation of the PI3K/AKT/mTOR pathway is frequently observed in multiple types of cancer, including brain, prostate, breast, lung, and liver cancer^25^,^26^,^27^,^28^. Thus, inhibiting mTOR signaling has become a viable and attractive option for molecular-targeted therapy in human cancers. The dual mTORC1/2 inhibitors, including PP242, INK128, AZD8055 and OSI-027, have demonstrated considerable efficacy in reducing tumor growth *in vivo* and *in vitro*^24^,^29^. In this study, the mTORC1/2 inhibitors PP242 and OSI-027 decreased the viability of H460 and A549 cells in a dose-dependent manner. However, apoptosis induction was negligible, as determined by annexin V staining and the evaluation of caspase 3/7 activity and PARP cleavage. These findings suggested that apoptosis does not contribute to the mTORC1/2 inhibitor-mediated decrease in NSCLC cell viability.

Extensive cellular vacuolation was observed in PP242-treated H460 and A549 cells. It was previously reported that vacuole accumulation is a morphological characteristic of autophagy^18^. Autophagy is targeted by some anticancer therapies. Autophagy can be activated by oxidative stress, nutrient deprivation and chemotherapy, and it promotes the degradation of damaged cytoplasmic proteins and organelles in response to stressors^30^. During autophagy, LC3-I is converted to the lipidated LC3-II form, which is considered a hallmark of autophagy and an essential requirement for the formation of the autophagosome^31^. The expression of autophagy-associated genes (Atg5, Atg7, and Beclin 1) generally reflects their autophagic activity^19^. p62 is used as a marker of autophagic flux, and p62 levels are inversely correlated with autophagic activity^20^. In this study, the mTORC1/2 inhibitor PP242 induced autophagy in NSCLC cells, as demonstrated by the formation of acidic vesicular organelles and the increase of LC3 puncta and LC3-II form. However, the protein levels of Atg5, Atg7, Beclin 1 and p62 were not affected by PP242.

Pre-treatment with the autophagy inhibitors 3-MA, CHX or BAF attenuated the formation of vacuoles induced by PP242. 3-MA or CHX, which block the initiation of autophagy, strongly reduced the PP242-induced increase in LC3-II levels. Knock-down of Atg5 or Atg7 by siRNAs also blocked LC3-II expression induced by PP242. However, treatment with BAF alone enhanced the accumulation of LC3-II. The increase in LC3-II levels in cells treated with a combination of PP242 and BAF was enhanced compared with cells treated with PP242 alone. LC3-II is degraded in lysosomes during autophagy; however, LC3-II can accumulate if protein degradation is inhibited. BAF disrupts intralysosomal degradation by inhibiting acidification^32^. Thus, LC3-II accumulates even under normal conditions, as the turnover of LC3-II mediated by basal levels of autophagy is blocked. These findings suggested that mTORC1/2 inhibitor enhances autophagic flux.

JNK participates in the autophagy induced by various types of stimuli, including nutritional deficiency, reductions in cytokines and growth factors, and neurotoxic drugs^33^,^34^,^35^,^36^. JNK activation has been shown to induce Bcl-2 phosphorylation, disrupt the Bcl-2/Beclin 1 complex, and promote the release of Beclin 1 to induce autophagy^33^. In this study, PP242 induced JNK activation (Fig. 4, Supplementary Fig. S1) but did not induce Bcl-2 phosphorylation in NSCLC cells (data not shown). Inhibiting JNK using SP600125 or siRNA reduced PP242-induced vacuole formation and LC3-II accumulation. These findings suggested that mTORC1/2 inhibitor-induced autophagy is mediated by JNK activation. In previous report^37^, an mTORC1 inhibitor, rapamycin, activates ASK1-JNK signaling and activation is dependent on the expression of 4E-BP1 suppressor proteins. In this study, PP242-induced JNK activation is not mediated by ASK1 (Supplementary Fig. S2). Further studies are needed to find out the mechanism of JNK regulation by mTORC1/2 inhibition.

The role of autophagy in promoting either cell survival or death is dependent on specific stimuli and cell types^38^. Autophagy induced by gefitinib primarily exerts cytotoxic effects on glioma cells, whereas gefitinib-induced autophagy in lung cancer cells exerts a cytoprotective effect^39^,^40^. In this study, inhibiting mTORC1/2 inhibitor-induced autophagy or JNK activation enhanced the mTORC1/2 inhibitor-induced reduction in cell viability, suggesting that mTORC1/2 inhibitor-induced autophagy exerted a cytoprotective effect in NSCLC cells.

In summary, mTORC1/2 inhibitors induced JNK activation and autophagy in NSCLC cells, and inhibiting JNK activation or autophagy strongly enhanced the mTORC1/2 inhibitor-induced decrease in NSCLC cell viability. These finding suggest that the combination of mTORC1/2 inhibitors with inhibitors of autophagy or JNK might be an effective approach for improving therapeutic outcomes in NSCLC.

## Materials and Methods

### Cell culture and reagents

The A549 and H460 lung cancer cell lines were obtained from the American Type Culture Collection (Manassas, VA, USA) and cultured in RPMI1640 medium (Welgene, Daegu, Republic of Korea) supplemented with 10% fetal bovine serum. Bafilomycin A1, cycloheximide, PP242, and 3-(4,5-dimethylthiazolyl-2)-2,5-diphenyltetrazolium bromide (MTT), and 3-methyladenine (3-MA), were purchased from Sigma-Aldrich (St. Louis, MO, USA), and OSI-027, PD98059, SB203580, and SP600125 were obtained from Selleck Chemicals (Houston, TX, USA).

### Measurement of cell viability

Cell viability was assessed by measuring the mitochondrial conversion of MTT. Cells treated with the indicated agents were incubated with the MTT reagent and solubilized in DMSO. The proportion of converted MTT was calculated by measuring absorbance at 570 nm. The results are expressed as the percent reduction in MTT, assuming that the absorbance of the control cells was 100%. The MTT experiments were repeated 3 times.

### Evaluation of apoptosis

Apoptosis was evaluated using the Annexin V-FITC Apoptosis Detection kit I according to manufacturer’s instructions (BD Biosciences, San Jose, CA, USA). Briefly, cells were collected, washed with cold PBS and suspended in annexin V binding buffer. The cells were stained with annexin V-FITC and subsequently analyzed using a FACScan flow cytometer (Becton Dickinson, San Jose, CA, USA). Each experiment was repeated 3 times.

### Measurement of caspase activation

Caspase activation was evaluated using a CaspaTag Caspase 3/7 *in situ* Assay Kit (Millipore, Billerica, MA, USA), according to the manufacturer’s instructions. The green fluorescence signal directly corresponds to the amount of active caspases present in the cells. Stained cells were analyzed using a FACScan flow cytometry platform.

### Quantification of acidic vacuoles

Cells treated with the indicated agents were trypsinized, collected, and stained with acridine orange for 10 min. After the cells were centrifuged, they were resuspended in PBS and analyzed using a FACScan flow cytometry platform.

### Immunofluorescence microscopy

Cells were grown on glass coverslips (Fisher Scientific, Pittsburgh, PA, USA) and treated in the presence or absence of PP242 for 6 h. Coverslips were incubated with anti-LC3 antibody (Sigma), followed by AlexaFluor 488 labeled anti-rabbit antibody (Thermo Fisher Scientific, Rockford, IL, USA). Next, coverslips were mounted with Vectashield mounting medium (Vector Laboratories, Burlingame, CA, USA) and analyzed via confocal microscopy (LSM-510, Carl Zeiss, Oberkochen, Germany).

### siRNAs and transfections

The JNK (#6232) and control (#6568) siRNAs were purchased from Cell Signaling Technology (Beverly, MA, USA). Atg5 (sequence: GGAAUAUCCUGCAGAAGAAdTdT)^41^ and Atg7 (sequence: GAAGAUAACAAUUGGUGUAUUdTdT)^41^, and control (sequence: CCUACGCCAAUUUCGUdTdT) siRNAs were synthesized by Bioneer (Daejeon, Republic of Korea). The transfection experiments were performed using Lipofectamine 2000 reagent according to the manufacturer’s instructions (Invitrogen, Carlsbad, CA, USA).

### Western blot analysis

The proteins in cell lysates were separated using SDS-PAGE and transferred to a PVDF membrane. The membrane was blotted with specific primary and horseradish peroxidase-conjugated secondary antibodies. The immunoreactive bands were visualized using SuperSignal West Pico Chemiluminescent Substrates (Thermo Fisher Scientific).

The following antibodies were used: Atg5 (#12994), Atg7 (#8558), Beclin 1 (#3738), cleaved PARP (#9541), JNK (#9252), p-Akt at Ser473 (#9271), p-c-Jun at Ser 63 (#9261), p-ERK at Thr202/Tyr204 (#9101), p-JNK at Thr183/Tyr185 (#9251), p-p38 at Thr180/Tyr182 (#9211), and p-S6 at Ser240/244 (#4838) were obtained from Cell Signaling Technology, p62/SQSTM1 (#H00008878-M01) from Abnova (Taipei City, Taiwan), and LC3 (L8918) and β-actin (#A5316) from Sigma-Aldrich.

### Statistical analysis

The data are presented as the mean ± standard deviation (SD) of 3 independent experiments. The statistical analyses were conducted using Student’s t-tests. A p-value less than 0.05 was considered statistically significant.

## Additional Information

**How to cite this article**: Jin, H.-O. *et al.* Inhibition of JNK-mediated autophagy enhances NSCLC cell sensitivity to mTORC1/2 inhibitors. *Sci. Rep.* **6**, 28945; doi: 10.1038/srep28945 (2016).

## Supplementary Material

Supplementary Information
