# Supplementary material for: Inhibition of JNK-mediated autophagy enhances NSCLC cell sensitivity to mTORC1/2 inhibitors
Source: Sci Rep. 2016 Jun 30;6:28945. doi: 10.1038/srep28945 (PMC4928093; doi:10.1038/srep28945)
Supplement: Supplementary Information [file srep28945-s1.pdf]

Supplementary figure 1.

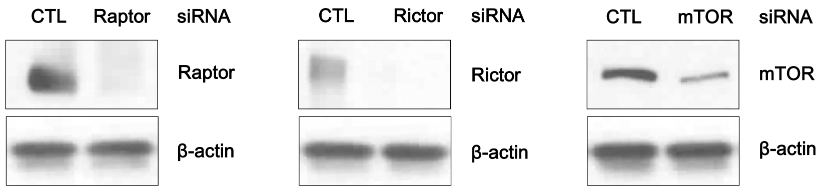

H460 were transfected with control, Raptor, Rictor, or mTOR siRNAs for 30 h. The indicated protein levels were measured via western blot analysis. CTL; control.

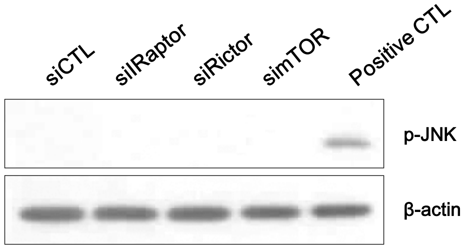

H460 were transfected with control, Raptor, Rictor, or mTOR siRNAs for 30 h. The indicated protein levels were measured via western blot analysis. Positive CTL; cell lysates prepared from H460 cells treated with 10 μM PP242 for 24 h. CTL; control.

Supplementary figure 2.

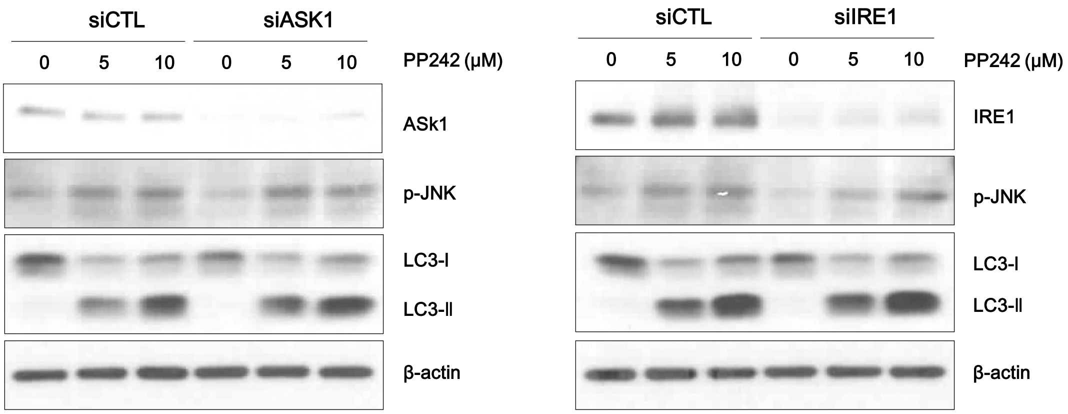

H460 cells were transfected with control, IRE1, or ASK1 siRNAs for 16 h, and then treated with the indicated concentrations of PP242 for 24 h. The indicated protein levels were measured via western blot analysis. CTL; control.

Supplementary figure 3.

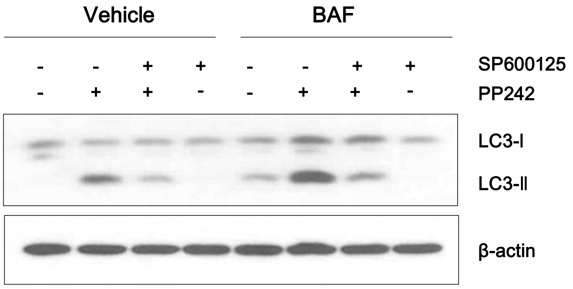

H460 cells were treated with 10 μM SP600125 and/or 10 μM PP242 for 20 h, followed by 100 nM bafilomycin A1 for 3 h. The indicated protein levels were measured via western blot analysis. BAF; bafilomycin A1.
